# Cost-effectiveness of a ketoanalogue-supplemented very low-protein diet in CKD

**DOI:** 10.1093/ndt/gfaf123

**Published:** 2025-07-08

**Authors:** Massimiliano Povero, Tatiana Simion, Lorenzo Pradelli

**Affiliations:** AdRes-Health Economics and Outcome Research, 10155 Turin, Italy; AdRes-Health Economics and Outcome Research, 10155 Turin, Italy; AdRes-Health Economics and Outcome Research, 10155 Turin, Italy

**Keywords:** chronic kidney disease, cost-effectiveness, ketoanalogue supplementation, low-protein diet, very low-protein diet

## Abstract

**Background:**

Chronic kidney disease (CKD) is a global health concern with significant economic implications, particularly in the management of end-stage renal disease (ESRD), which often requires kidney replacement therapy such as dialysis or transplantation. The rising incidence of ESRD is expected to increase healthcare costs substantially by 2030. Dietary protein restriction is a cornerstone of managing CKD progression, and the use of ketoanalogues in very low-protein diets (VLPD) has shown promise in delaying dialysis initiation. This study evaluates the cost-effectiveness of a vegetarian ketoanalogue-supplemented VLPD (s-VLPD) compared with a low-protein diet (LPD) in patients with CKD stages 4 and 5 in Italy.

**Methods:**

A Markov model was developed to simulate the health outcomes and costs for patients with CKD stages 4 or 5, treated with either s-VLPD or LPD. The analysis was conducted from both the Italian National Healthcare Service (NHS) and societal perspectives. Healthcare costs considered were: ketoanalogue supplementation, dialysis and diet monitoring. Overall survival and quality-adjusted life years (QALYs) were used as effectiveness outcomes. Sensitivity analyses, including deterministic and probabilistic approaches, assessed the robustness of the results.

**Results:**

The s-VLPD strategy led to improved survival (+0.60 years) and increased QALYs (+0.49) compared with LPD, along with cost savings of approximately €34 000 per patient from the NHS perspective. From the societal perspective, s-VLPD resulted in a cost saving of €59 147 per patient. Sensitivity analyses confirmed that s-VLPD remains a dominant strategy, demonstrating both clinical and economic advantages.

**Conclusion:**

s-VLPD is a cost-effective strategy for managing CKD stages 4 and 5, offering improved patient outcomes and significant cost savings. The findings support the integration of s-VLPD in routine clinical practice, helping delay dialysis initiation, reduce the financial burden on healthcare systems and improve patient quality of life.

KEY LEARNING POINTS **What was known:**

- Chronic kidney disease (CKD) is a global health burden, with end-stage cases requiring costly dialysis or transplantation.
- A very low protein diet supplemented with ketoanalogues (s-VLPD) can delay dialysis initiation.
- Although previous studies have suggested potential benefits of a ketoanalogue s-VLPD, its cost-effectiveness remains uncertain compared with a standard low-protein diet (LPD).

**This study adds:**

- s-VLPD delays dialysis initiation, improves survival and enhances quality-adjusted life years compared with LPD.
- From both the Italian National Healthcare Service and societal perspectives, s-VLPD is cost-saving and reduces healthcare expenses.
- Sensitivity analyses confirm s-VLPD as a dominant, cost-effective strategy for CKD management.

**Potential impact:**

- Integrating ketoanalogue s-VLPD into routine CKD management can reduce the financial burden on healthcare systems by delaying the need for dialysis.
- Patients benefit from improved quality of life and extended survival, with fewer complications associated with late-stage CKD.

## INTRODUCTION

Chronic kidney disease (CKD) is a pervasive condition and represents a significant driver of global healthcare expenses [1, 2]. The economic impact of CKD stems largely from the high costs associated with managing end-stage renal disease (ESRD), which necessitates kidney replacement therapy (KRT) such as dialysis or transplantation [1]. By 2030, the global population requiring KRT is estimated to rise substantially, from 2.6 million to over 5.4 million individuals [3]. KRT is not only expensive but also imposes a substantial burden on patients, their families and society. Consequently, interventions that delay the progression to KRT can potentially alleviate the economic strain caused by CKD-related healthcare expenditures and productivity losses [4].

CKD is characterized by the reduced ability of kidneys to eliminate the waste products of protein metabolism, leading to an accumulation of uraemic toxins in the blood and increased glomerular hyperfiltration [5, 6]. Restricting dietary protein intake is a key component of conservative treatment in pre-dialysis CKD patients. Ketoanalogues are nitrogen-free compounds that mimic essential amino acids, allowing their conversion into amino acids through endogenous transamination, and enable protein synthesis while minimizing the production of toxic nitrogenous waste [7]. Supplementing protein-restricted diets with ketoanalogues reduces nitrogen consumption without the adverse effects of a severely low-protein diet, as long as adequate energy intake is maintained [7].

For adults with CKD stages 3 to 5 who are metabolically stable, the Kidney Disease Outcomes Quality Initiative (KDOQI) guidelines recommend dietary protein restriction with or without ketoacid analogues to reduce the risk of ESRD and mortality, while improving quality of life (QoL). These recommendations include a low-protein diet (LPD) providing 0.6 g/kg/day of dietary protein, or a very low-protein diet (VLPD) providing 0.3–0.4 g/kg/day supplemented with ketoanalogues to meet protein requirements (0.6 g/kg/day) [5]. The more recent KDIGO 2024 guidelines provide more specific recommendations regarding dietary protein management in patients with CKD stages 4+ without relevant comorbidities [5].

A 2016 randomized controlled trial, followed by a long-term study, demonstrated that a vegetarian ketoanalogue-supplemented VLPD (s-VLPD; 0.3 g/kg/day plus 1 tablet of Ketosteril per 5 kg of ideal body weight/day) effectively delayed dialysis initiation compared with an LPD (0.6 g/kg/day) in nondiabetic patients with CKD stages 4 and 5 [8, 9].

These findings also suggested a potential reduction in overall costs (both direct and indirect costs) [10]. Th aim of this study was to evaluate the cost-effectiveness of s-VLPD compared with LPD alone in patients with CKD stages 4 or 5 from both the Italian National Healthcare Service (NHS) and societal perspectives.

## MATERIALS AND METHODS

### Model design

**Figure 1:**
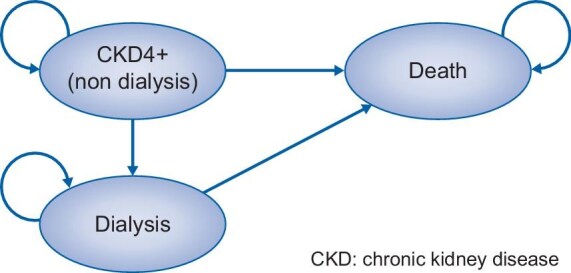
Markov model scheme.

A Markov model was developed to assess health outcomes and costs for patients with CKD stages 4 or 5 in Italy who were treated with either a VLPD (0.3–0.4 g/kg/day) with ketoanalogue supplementation (4–8 tablets, three times daily), or LPD (0.6 g/kg/day). All patients entered the model in the pre-dialysis stage [estimated glomerular filtration rate (eGFR) <30 mL/min/1.73 m²]. At each model cycle, patients could remain in the pre-dialysis health state, start dialysis treatment or die. The model structure illustrated in Fig. 1 was the same for the two strategies considered, while transition probabilities and costs are specific for each strategy. Disease progression was simulated across the patients’ lifetime, with each model cycle representing 1 month.

As a base case, the analysis was conducted from the Italian NHS perspective, including the cost of drugs, dietary monitoring and dialysis. The societal perspective was also considered as a scenario analysis, including costs related to productivity losses and caregiver expenses. By health economic recommendations [11], the model applied a 3% discount rate to future costs and health outcomes. The incremental cost-effectiveness ratio was used to compare s-VLPD versus LPD, calculated as the cost difference divided by the difference in quality-adjusted life years (QALYs).

### Clinical input

Age and gender distribution of CKD stages 4 or 5 patients in Italy were derived from a population of approximately 100 000 individuals identified in the Lazio Region [12]. Men represented 55.8% of the cohort, with a mean age of 70 years for males and 72 years for females.

The annual probability of progressing from the CKD stages 4 or 5 states to dialysis in the LPD arm was 24.4% [8]. In contrast, for the s-VLPD arm, the onset of dialysis was estimated based on the adjusted hazard ratio (HR) derived from the same study [8] [HR 0.237, 95% confidence interval (CI) 0.22 to 0.26].

Mortality in CKD stages 4 or 5 patients before initiating dialysis was estimated from a prospective observational study conducted in patients following a moderately restricted LPD at the Nephrology Unit of San Luigi Hospital, Turin, between 2007 and 2015 [13]. Annual mortality for CKD patients was found to be 11.1%.

Annual dialysis-related mortality was estimated at 13.8% based on a retrospective observational study of 487 patients treated at the Nephrology Unit of Santa Chiara Hospital, Trento, between 2008 and 2014, with follow-up until 2015 [14].

All baseline and clinical input are reported in Supplementary data, Table S1.

### Utilities

The time spent in each health state is assigned a corresponding utility value. A thorough review of internationally published literature was carried out to identify relevant utility estimates for patients with CKD stages 4 and 5 who are not yet on dialysis and for those undergoing dialysis.

Four key studies were selected from the various sources examined to inform the cost-utility model. The meta-analysis by Wyld and colleagues [15] was chosen as the base-case reference, as it provided utility values specific to different dialysis modalities.

### Cost inputs

The model accounted for the costs of ketoanalogue supplementation (for the VLPD strategy), additional supplements, diet monitoring and dialysis, using locally derived data. The cost of ketoanalogues supplementation was calculated considering the ex-factory price of Ketosteril (box of 100 tablets) and the average posology for adults (6 tablets three times daily) [16] (Supplementary data, Table S1).

Further supplementations included sodium bicarbonate, vitamin D and calcium as the only supplements showing a statistically significant variation between s-VLPD and LPD in the Garneata *et al*. trial [9] (Supplementary data, Table S1). Specifically, sodium bicarbonate was used by 51% of patients on LPD with an average dosage of 6.4 tablets per day, whereas only 29% of s-VLPD patients required it, with a lower daily intake of 4.4 tablets. Vitamin D supplementation was taken by 54% of LPD patients at a dosage of 1 tablet per week, while only 22% of s-VLPD patients required it, with the same intake. Similarly, calcium supplementation was used by 50% of patients in both groups, but the dosage differed slightly, with LPD patients taking 6.9 tablets per day compared with 6.3 tablets per day in the s-VLPD group.

Diet monitoring costs were estimated as one consultation per month for CKD stages 4 or 5 patients on VLPD and one consultation every 2 months for those on LPD (assumed less frequent due to less dietary restriction) (Supplementary data, Table S1).

Finally, dialysis cost was calculated as the mean of haemodialysis (HD), continuous ambulatory peritoneal dialysis (CAPD) and automated peritoneal dialysis (APD) outpatient service tariffs [17] (Supplementary data, Table S1), weighted for the corresponding frequencies reported in the 2019 Italian Census [18] (83.8% for HD, 7.7% for CAPD and 8.5% for APD). Additional one-off costs for PD included catheter placement and annual maintenance (Supplementary data, Table S1).

### Sensitivity analysis

Sensitivity analysis tested the robustness of the cost-effectiveness results from the payer's perspective. Deterministic sensitivity analysis evaluated uncertainty in individual input parameters by varying them one at a time while holding others constant. Costs and dialysis distribution varied by ±20% of the base case, while clinical efficacy and QoL parameters varied within their 95% CI limits.

A probabilistic sensitivity analysis (PSA) was conducted to evaluate confidence in the results given input uncertainties. Parameters were randomly sampled from predefined distributions (Supplementary data, Table S1), and the model was run 1000 times. Outputs were analysed on the cost-effectiveness plane to produce a distribution of results.

### Scenario analysis

As an alternative analysis, a societal perspective was included, accounting for indirect costs such as productivity losses and caregiver expenses.

The cost of time lost due to dialysis was calculated based on the monthly value of paid and unpaid work (household tasks, caregiving and volunteering) [19], which was stratified by age and gender (Supplementary data, Table S1). This value was then multiplied by the proportion of time lost for each dialysis type, assumed to be equal to 50% of the week for HD, 30% for CAPD and 20% for APD.

Caregiver costs were estimated using the same monthly value of paid and unpaid work, considering that 63.3% of caregivers were employed [20], with 58% being male and a mean age of 51.7 years [21]. Additionally, 24.5% of patients in PD required caregiver assistance [18], and the same proportion has been assumed for patients in HD. The weekly hours of care required were estimated to be 12 h for HD (accounting for three hospital visits per week) and 2 h for PD (assuming minimal support due to overnight dialysis).

Two further scenario analyses were implemented in order to investigate the impact of model assumptions on results. First, as it is reasonable to expect that s-VLPD not only delays the need for dialysis but may also help maintain patients in relatively better health after dialysis initiation, the conservative assumption of same mortality risk in dialysis (among both patients treated with LPD and s-VLPD in the pre-dialysis state) has been weakened by assuming a reduction in the risk of mortality in dialysis for patients previously treated with s-VLPD. Such reduction was estimated based on a retrospective study that examined patients starting dialysis between 2001 and 2013 who received more than 3 months of LPD supplemented with Ketosteril in the year preceding the start of dialysis [22]. Patients showed an all-cause mortality reduction (HR 0.77, 95% CI 0.70–0.84) with respect to patients without keto analogous supplementation. Based on the estimated reduction, in this scenario analysis the mortality in dialysis for patients in the s-VLPD arm has been reduces to 10.8% instead of 13.8% as in the base case.

Second, as highlighted in the last guideline [5], the adherence to protein-restricted diet could be difficult and lower than that observed in the trials by Garneata *et al*. [8, 9]. In the base-case analysis, full adherence (100%) to the s-VLPD regimen was assumed; however, to reflect more realistic clinical conditions, a sensitivity analysis was performed considering partial adherence. Specifically, it was assumed that 42% of patients initially assigned to the s-VLPD group were unable to maintain the diet and transitioned to an LPD within the first 6 months of treatment. This transition was modeled as a linear redistribution of patients from the s-VLPD group to the LPD group over that period. Patients switching to LPD lose the clinical benefit and their risk of starting dialysis was assumed, from then on, equal to the risk of patients in the LPD arm.

## RESULTS

### Cost-effectiveness results

s-VLPD leads to both improved survival (+0.60 years) and enhanced quality-adjusted life years (0.49 QALYs), alongside cost savings of nearly €34 000 (in the NHS perspective), making it a dominant strategy compared with LPD (Table 1). The clinical and economic advantages observed in patients treated with s-VLPD are primarily attributed to the substantial delay in the initiation of dialysis (+2.91 years) and the extended survival (+0.60 years). In the scenario analysis based on the societal perspective, the total cost of care per patient was estimated at €139 714 for LPD and €80 566 for s-VLPD, resulting in a cost difference of –€59 147 per patient. As with the NHS perspective, s-VLPD emerges as a dominant strategy compared with LPD.

**Table 1:** Base case results.

	LPD	s-VLPD	Delta s-VLPD vs LPD
Survival (years)	7.27	7.87	0.60
Time pre-RRT	2.54	5.44	2.91
Time in dialysis	4.74	2.43	–2.31
QALYs	4.45	4.94	0.49
Total costs (NHS perspective)	€91 445.37	€57 603.38	–€33 841.99
Ketoanalogous	€0.00	€12 432.94	€12 432.94
Diet monitoring	€312.26	€1 240.98	€928.72
Dialysis	€90 578.71	€43 029.43	–€47 549.28
Other supplementation	€554.40	€900.03	€345.63
Total costs (societal perspective)	€139 713.51	€80 566.35	–€59 147.16
Keto analogous	€0.00	€12 432.94	€12 432.94
Diet monitoring	€312.26	€1 240.98	€928.72
Dialysis	€90 578.71	€43 029.43	–€47 549.28
Other supplementation	€664.39	€985.47	€231.08
Indirect costs	€48 158.15	€22 877.53	–€25 280.61

NHS, Italian National Healthcare Service; RRT, renal replacement therapy.

### Deterministic sensitivity analyses

**Figure 2:**
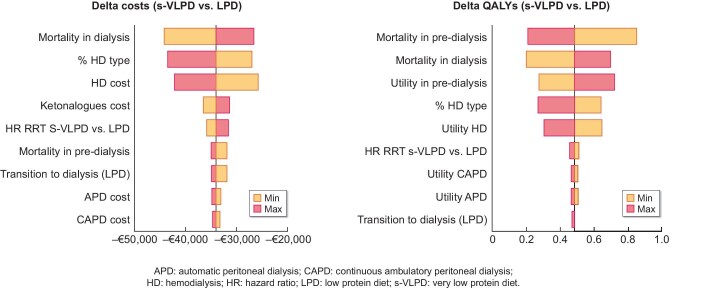
Tornado diagrams of incremental costs (left) and incremental efficacy (right) resulting from deterministic sensitivity analysis.

A deterministic (one-way) sensitivity analysis was carried out to address the uncertainty in the differences between costs and QALYs by varying input parameters within predefined ranges. Even when using the most conservative assumptions for the input values, s-VLPD continues to demonstrate greater effectiveness and lower costs compared with LPD, confirming its dominance (Fig. 2).

### Probabilistic sensitivity analysis

**Figure 3:**
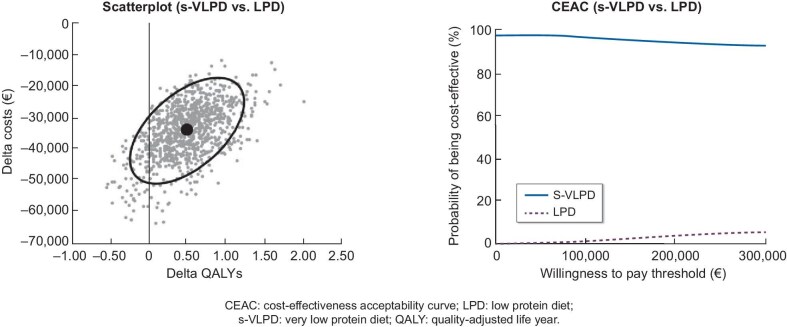
Results of probabilistic sensitivity analysis: incremental cost-effectiveness plane (left) and cost-effectiveness acceptability curve (right). CEAC, cost-effectiveness acceptability curve.

The PSA results align with the deterministic findings, confirming the conclusions of the base case (Fig. 3). The estimated savings resulting from adding Ketosteril to VLPD range between €18 000 and €49 000, while the QALY gains from –0.26 to 1.25. The ellipse delineates the 95% CI, identifying the region where the ‘true’ cost-utility analysis (CUA) result is most likely to fall, predominantly within the quadrant of dominance [Fig. 3(left)].

Figure 3 (right) shows the cost-effectiveness acceptability curve. At any willingness-to-pay thresholds, s-VLPD demonstrates a nearly 100% probability of being cost-effective compared with LPD.

### Scenario analysis results

In the scenario analysis assuming a long-term mortality benefit of s-VLPD, patients treated with s-VLPD experienced greater benefits of LPD patients than in the base case (Supplementary data, Table S2). Specifically, the gain in survival and quality of life increased to 1.30 years and 0.78 QALY, respectively. Due to the reduction in mortality in dialysis, the average time spent in dialysis for patients treated with s-VLPD increase from 2.43 of the base case to 3.13 years. s-VLPD still remained dominant with respect to LPD strategy even if the saving in dialysis costs was slightly lower.

When accounting for less than full compliance with the s-VLPD regimen, the strategy remained more effective (survival +0.25 years and quality of life +0.21 QALY) and less costly (–€10 977.42), despite a proportion of patients switching diets during the initial treatment phase (Supplementary data, Table S2).

## DISCUSSION

The study evaluated the cost-effectiveness of s-VLPD versus LPD in patients with CKD stages 4 or 5 in Italy from both the NHS and societal perspectives. Results demonstrated that the upfront investment for ketoanalogue supplementation in VLPD was completely offset by the long-term economic benefits. A key driver of this economic advantage is the delay in dialysis initiation (+2.91 years), which translates into a reduction in dialysis-related costs by €47 549. While ketoanalogue supplementation incurs an additional €12 433 and diet monitoring costs increase by €928.72, these expenses are more than offset by the reduced need for dialysis and related hospitalizations.

The scenario analyses suggest that s-VLPD may offer significant clinical and economic benefits, even under alternative assumptions of long-term mortality benefit and partial patient adherence, reinforcing its potential as a cost-effective strategy in CKD stages 4 or 5 management.

Sensitivity analyses confirmed the robustness of these findings, demonstrating that s-VLPD remains cost-effective across varying assumptions of costs and clinical efficacy. These results support the adoption of s-VLPD as a cost-effective strategy for managing patients with CKD, balancing both economic and clinical benefits.

A Ketosteril-supplemented VLPD showed the same good cost-effectiveness profile in previous analyses, with different studies demonstrating both clinical benefits and economic advantages. Cost-effectiveness analyses conducted in Vietnam, Taiwan, Thailand, Kazakhstan and Hungary [23–27] reported increased QALYs and healthcare cost savings when ketoanalogues supplementation was used alongside VLPD or LPD. These findings highlight the potential of s-VLPD to delay dialysis initiation, reduce the overall financial burden on healthcare systems and improve patient outcomes. The consistency of these results across different healthcare settings reinforces the economic viability of integrating s-VLPD into routine clinical practice for the management of pre-dialysis CKD patients.

The present analysis has several strengths, including the use of a validated model structure, the incorporation of country-specific cost data and resource utilization, and a conservative approach to assessing the cost-effectiveness of the intervention.

However, certain limitations should be considered when interpreting the findings. First, the mortality estimates for CKD stage 4 or 5 patients before initiating dialysis, derived from a prospective observational study conducted at the Nephrology Unit of San Luigi Hospital, Turin (2007–15). Although not all participants were in CKD stages 4 or 5, the median baseline eGFR was 20 mL/min/1.73 m², and mortality rates for those with eGFR <15 or <10 mL/min/1.73 m² were only slightly higher than the overall cohort. These findings suggest that even in patients with more advanced CKD, dietary interventions may help manage disease progression without a substantial increase in mortality risk. However, since the study population followed a moderately restricted LPD rather than an s-VLPD regimen, its direct applicability to the present analysis remains limited. Second, due to the lack of country-specific clinical data, key model inputs, such as health state transition probabilities and QoL outcomes, were derived from a 15-month randomized controlled trial with long-term follow-up (120 months) conducted in a specialized nephrology center in Romania. The study population consisted of carefully selected adults with stable eGFR <30 mL/min/1.73 m², proteinuria <1 g/g urinary creatinine, good nutritional status and strong adherence to dietary recommendations, excluding patients with diabetes. Additionally, all participants underwent a 3-month run-in phase on LPD before randomization to LPD or s-VLPD. As a result, the findings of that study and its follow-up are only generalizable to a similar population in Italy.

Finally, the cost analysis primarily considered direct medical expenses, including those for ketoanalogues, sodium bicarbonate, vitamin D and calcium supplements, dietary monitoring and dialysis. While the societal and patient perspective analysis partially accounted for indirect costs such as dialysis-related complications, home modifications, medical supplies, patient transportation, unpaid caregiver time and productivity loss certain elements remained unquantified. A more comprehensive inclusion of these costs would likely strengthen the case for s-VLPD's cost-effectiveness by further emphasizing its potential to alleviate both the financial and practical burdens on patients and their families.

In conclusion, although published literature on s-VLPD remains limited, the available evidence strongly supports its concurrent clinical and economic benefits. Simulation models, based on clinical outcomes from existing studies and country-specific cost data, demonstrate that s-VLPD is a dominant strategy, offering both greater effectiveness and lower costs compared with LPD in patients with CKD stages 4 or 5. Given the rising prevalence of ESRD and the substantial financial burden of renal replacement therapy, these findings reinforce the importance of healthcare policies aimed at delaying dialysis initiation, including the appropriate use of s-VLPD in suitable CKD stages 4 or 5 patients.

## Supplementary Material

gfaf123_Supplemental_File

## Data Availability

The data underlying this article are available in the article and in its online supplementary material.
