# Supplementary material for: Cost-effectiveness of a ketoanalogue-supplemented very low-protein diet in CKD
Source: Nephrol Dial Transplant. 2025 Jul 8;40(12):2372–8. doi: 10.1093/ndt/gfaf123 (PMC12648053; doi:10.1093/ndt/gfaf123)
Supplement: gfaf123_Supplemental_File [file gfaf123_supplemental_file.docx]

## Additional Material

Table S1. Base case input

| **Variables** | **Base value** | **SE/95% CI** | **Distribution used in PSA** | **Source** |
| --- | --- | --- | --- | --- |
| **Baseline characteristics** |  |  |  |  |
| Age (years) | 70.9 | SE= 10% of mean | Normal | Marino 2020^13^ |
| Gender (% female) | 44.2% | SE= 10% of mean | Normal* | Marino 2020^13^ |
| **Clinical input** |  |  |  |  |
| Freq. Use – HD over HD+PD | 83.8% | SE= 10% of mean | Normal* | Neri 2022^19^ |
| Freq. Use – CAPD over CAPD+APD | 47.3% | SE= 10% of mean | Normal* | Neri 2022^19^ |
| Annual dialysis onset (LPD) | 24.4% | SE= 10% of mean | Normal* | Neri 2022^19^ |
| Annual mortality pre-dialysis | 11.1% | SE= 10% of mean | Normal* | Neri 2022^19^ |
| Annual mortality in dialysis | 13.8% | SE= 10% of mean | Normal* | Rigoni 2017^15^ |
| HR dialysis onset (s-VLPD vs. LPD) | 0.24 | 0.22 to 0.26 | Lognormal | Garneata 2019^9^ |
| **Utility** |  |  |  |  |
| QoL CKD-4+ | 0.790 | 0.70 to 0.89 | Normal | Wyld 2012^16^ |
| QoL HD | 0.690 | 0.59 to 0.80 | Normal | Wyld 2012^16^ |
| QoL CAPD | 0.720 | 0.60 to 0.85 | Normal | Wyld 2012^16^ |
| QoL APD | 0.800 | 0.69 to 0.91 | Normal | Wyld 2012^16^ |
| **Cost input** |  |  |  |  |
| Ketosteril (cost per 100 tablets) | € 40.23 | SE= 10% of mean | Normal | L’Informatore Farmaceutico 2024^17^ |
| Specialistic visit (diet monitoring) | € 22.00 | SE= 10% of mean | Normal | L’Informatore Farmaceutico 2024^17^ |
| Sodium box 1000 tablets | € 39.00 | SE= 10% of mean | Normal | L’Informatore Farmaceutico 2024^17^ |
| Vit D box 4 tablets | € 6.32 | SE= 10% of mean | Normal | L’Informatore Farmaceutico 2024^17^ |
| Calcium box 30 tablets | € 4.52 | SE= 10% of mean | Normal | L’Informatore Farmaceutico 2024^17^ |
| HD (cost per session) | € 159.31 | SE= 10% of mean | Normal | 2024 Italian Outpatient Service Tariff^18^ |
| APD | € 64.40 | SE= 10% of mean | Normal | 2024 Italian Outpatient Service Tariff^18^ |
| CAPD | € 51.98 | SE= 10% of mean | Normal | 2024 Italian Outpatient Service Tariff^18^ |
| Catheter placement | € 179.60 | SE= 10% of mean | Normal | 2024 Italian Outpatient Service Tariff^18^ |
| Catheter maintenance annual | € 46.20 | SE= 10% of mean | Normal | 2024 Italian Outpatient Service Tariff^18^ |
| Paid work male age 65+ | € 264,00 | SE= 10% of mean | Normal | Pradelli 2017^20^ |
| Paid work female age 65+ | € 42,25 | SE= 10% of mean | Normal | Pradelli 2017^20^ |
| Unpaid work male age 65+ | € 544,25 | SE= 10% of mean | Normal | Pradelli 2017^20^ |
| Unpaid work female age 65+ | € 1.207,58 | SE= 10% of mean | Normal | Pradelli 2017^20^ |

APD: automatic peritoneal dialysis; CAPD: continuous ambulatory peritoneal dialysis; CI: confidence interval; CKD: chronic kidney disease; DSA: deterministic sensitivity analysis; HD: hemodialysis; HR: hazard ratio; PD: peritoneal dialysis; PSA: Probabilistic sensitivity analysis; QoL: quality of life.

* In the PSA, the proportion p was normalized using the logit transformation i.e., logit(p)=log(p/(1-p)) and then the values of logit(p) were sampled from a normal distribution; sampled values were then re-converted into probabilities using the inverse sigmoid function s(x)=exp(x)/(1+exp(x)).

Table S2. Scenario analyses results

| **Long-term benefit of s-VLPD on mortality** | | | |
| --- | --- | --- | --- |
|  | **LPD** | **s-VLPD** | **Delta s-VLPD vs. LPD** |
| **Survival (years)** | **7.27** | **8.57** | **1.30** |
| Time pre RRT | 2.54 | 5.44 | 2.91 |
| Time in dialysis | 4.74 | 3.13 | -1.61 |
| Number of dialysis sessions | 902 | 596 | -306 |
| Number of monitoring visits | 15 | 65 | 50 |
| **QALYs** | **4.45** | **5.23** | **0.78** |
| **Total costs (NHS Perspective)** | **€ 91,445.37** | **€ 67,664.90** | **-€ 23,780.47** |
| Keto analogous | € 0.00 | € 12,432.94 | € 12,432.94 |
| Diet monitoring | € 312.26 | € 1,240.98 | € 928.72 |
| Dialysis | € 90,578.71 | € 53,090.95 | -€ 37,487.76 |
| Other supplementation | € 554.40 | € 900.03 | € 345.63 |
| **Total costs (Societal Perspective)** | **€ 139.713,51** | **€ 95.978,42** | **-€ 43.735,09** |
| Keto analogous | € 0.00 | € 12,432.94 | € 12,432.94 |
| Diet monitoring | € 312.26 | € 1,240.98 | € 928.72 |
| Dialysis | € 90,578.71 | € 53,090.95 | -€ 37,487.76 |
| Other supplementation | € 664.39 | € 985.47 | € 321.08 |
| Indirect costs | € 48,158.15 | € 28,228.08 | € -19,930.06 |
| **VLPD compliance** | | | |
|  | **LPD** | **s-VLPD** | **Delta s-VLPD vs. LPD** |
| **Survival (years)** | **7.27** | **7.52** | **0,25** |
| Time pre RRT | 2.54 | 3.73 | 1.20 |
| Time in dialysis | 4.74 | 3.79 | -0.95 |
| Number of dialysis sessions | 902 | 721 | -181 |
| Number of monitoring visits | 15 | 45 | 30 |
| **QALYs** | **4.45** | **4.66** | **0.21** |
| **Total costs (NHS Perspective)** | **€ 91,445.37** | **€ 80,467.96** | **-€ 10,977.42** |
| Keto analogous | € 0.00 | € 8,930.12 | € 8,930.12 |
| Diet monitoring | € 312.26 | € 891.35 | € 579.09 |
| Dialysis | € 90,578.71 | € 70,000.03 | -€ 20,578.68 |
| Other supplementation | € 554.40 | € 646.46 | € 92.06 |
| **Total costs (Societal Perspective)** | **€ 139,713.51** | **€ 117.746.37** | **-€ 21,967.14** |
| Keto analogous | € 0.00 | € 8,930.12 | € 8,930.12 |
| Diet monitoring | € 312.26 | € 891.35 | € 579.09 |
| Dialysis | € 90,578.71 | € 70,000.03 | -€ 20,578.68 |
| Other supplementation | € 664.39 | € 707.83 | € 43.44 |
| Indirect costs | € 48,158.15 | € 37,217.04 | € -10,941.11 |

LPD: low protein diet; NHS: 0Italian National Healthcare Service; QALY: quality-adjusted life year; RRT: renal replacement therapy; s-VLPD: supplemented very low protein diet.
